# A systematic review of reasons for and against asking patients about their socioeconomic contexts

**DOI:** 10.1186/s12939-019-1014-2

**Published:** 2019-07-23

**Authors:** Andrew Moscrop, Sue Ziebland, Nia Roberts, Andrew Papanikitas

**Affiliations:** 1 Nuffield Department of Primary Care Health Sciences, University of Oxford, Oxford, UK; 2 Nuffield Department of Population Health, University of Oxford, Oxford, UK

**Keywords:** Social determinants of health, Socioeconomic factors, Medical records

## Abstract

**Background:**

People’s social and economic circumstances are important determinants of their health, health experiences, healthcare access, and healthcare outcomes. However, patients’ socioeconomic circumstances are rarely asked about or documented in healthcare settings. We conducted a systematic review of published reasons for why patients’ socioeconomic contexts (including education, employment, occupation, housing, income, or wealth) should, or should *not*, be enquired about.

**Methods:**

Systematic review of literature published up to and including 2016. A structured literature search using databases of medicine and nursing (pubmed, embase, global health), ethics (Ethicsweb), social sciences (Web of Science), and psychology (PsychINFO) was followed by a ‘snowball’ search. Eligible publications contained one or more reasons for: asking patients about socioeconomic circumstances; collecting patients’ socioeconomic information; ‘screening’ patients for adverse socioeconomic circumstances; or linking other sources of individual socioeconomic data to patients’ healthcare records. Two authors conducted the screening: the first screened all references, the second author screened a 20% sample with inter-rater reliability statistically confirmed. ‘Reason data’ was extracted from eligible publications by two authors, then analysed and organised.

**Results:**

We identified 138 eligible publications. Most offered reasons for why patients’ *should* be asked about their socioeconomic circumstances. Reasons included potential improvements in: individual healthcare outcomes; healthcare service monitoring and provision; population health research and policies. Many authors also expressed concerns for improving equity in health. Eight publications suggested patients should *not* be asked about their socioeconomic circumstances, due to: potential harms; professional boundaries; and the information obtained being inaccurate or unnecessary.

**Conclusions:**

This first summary of literature on the subject found many published reasons for why patients’ social and economic circumstances *should* be enquired about in healthcare settings. These reasons include potential benefits at the levels of individuals, health service provision, and population, as well as the potential to improve healthcare equity. Cautions and caveats include concerns about the clinician’s role in responding to patients’ social problems; the perceived importance of social health determinants compared with biomedical factors; the use of average population data from geographic areas to infer the socioeconomic experience of individuals. Actual evidence of outcomes is lacking: our review suggests hypotheses that can be tested in future research.

## Background

Individual social and economic factors are known to be important determinants of a person’s health, [1] health experiences, [2] healthcare access, and healthcare outcomes. [3–5] Yet in many healthcare contexts there is no routine and systematic assessment of individual patients’ social and economic circumstances, including their level of education, employment status, occupation, housing status, or household income.

In the UK, where our study team is based, researchers have described a ‘social gradient in health’. [6] According to this gradient, those who are better-off, better educated, or from higher social classes are likely to live longer and healthier lives, while their less privileged contemporaries die sooner and suffer more from poor health while they are alive. Doctors working in UK general practice (primary care) routinely observe the social gradient in health within their practice population and witness the detrimental impact of adverse social circumstances on the health of individuals. [7] Meanwhile, patients frequently attend general practice with problems relating to welfare benefits, housing, or unemployment, and general practitioners (GPs) spend significant amounts of time discussing these non-medical issues with their patients. [8] A performance management scheme has been introduced to incentivise collection of certain patient data (including ethnicity) in UK general practice, yet currently no socioeconomic information is included in the routinely-collected patient data set. [9] Instead, clinical enquiries about ‘social’ factors, and hence the ‘social histories’ documented in patients’ clinical records, tend to halt at socially-influenced behaviours such as diet, exercise, alcohol consumption, and smoking habits rather than the socio-economic variables that underpin these behaviours. Even when socioeconomic information about patients is required for monitoring, service evaluation, research or other purposes, it is likely to be inferred from postal codes and the demographics of geographic areas, rather than using individual level data. The problems with this reliance on area data will be discussed below.

While the UK may not represent an average or norm, with its publicly-funded health and social care system and very high levels of income inequality, the issues around socioeconomic data collection in healthcare settings may be common to many countries and are being addressed in some contexts.

In America, the Obama-era healthcare reforms were intended to extend health insurance coverage to millions of previously un-insured Americans. Many of these people were on low incomes and often experiencing social deprivation. It was recognised that identifying and addressing their social needs would likely improve their health outcomes while restraining overall healthcare spending. [10] Legislation financially incentivised the ‘meaningful use’ of electronic health records and this included their use in recording patients’ social and behavioural health determinants. [11] The US Institute of Medicine (now the National Academy of Medicine) has delivered specific recommendations on which health determinants should be assessed during healthcare encounters and how the information should be measured and recorded. [12] The specified health determinants include: individual level of education and overall experience of financial resource strain. [13]

In Canada, around the same time as the developments in America, a group of healthcare practitioners and researchers in Toronto recognised that affordability or insurance cover are not the only barriers to healthcare. Even within Canada’s publicly-funded healthcare system, poverty can be a barrier to accessing care. If poverty, and other socio-economic characteristics, were routinely collected their associations with healthcare access and outcomes could provide an improved basis for more equitable care. Four large health organisations in Toronto have introduced routine collection of patients’ social data, including housing information and household income. [14]

Recognising the apparent inconsistency between the acknowledged importance of social health determinants and their absence in routinely collected UK health data, and recognising also the efforts to change data collection practices in other parts of the world, we felt prompted to conduct the current study. Using the method of a Systematic Review of Reasons, [15] we set out to answer the question: What reasons have been given for asking, or not asking, patients about their socioeconomic circumstances in healthcare settings? Our aim is to produce the first systematic review of the subject, summarising the published literature, including evidence and argument, while revealing points of contention or uncertainty, to inform practice, policy and research..

## Methods

We used a structured literature search, with initial title and abstract screening, followed ny whole-text review of the screened literature, and subsequent ‘snowballing’ from the citations to capture as much relevant material as possible. This was followed by the extraction and organisation of ‘reason data’ from eligible publications.

Full details of our study protocol and search strategy are contained in an appendix.

### Inclusion and exclusion criteria

Before searching, we agreed eligible publications must contain one or more stated reasons for or against undertaking the following activities in healthcare settings:

- asking patients about socioeconomic circumstances;
- collecting patients’ socioeconomic information;
- ‘screening’ patients for adverse socioeconomic circumstances;
- linking other sources of individual socioeconomic data (e.g. census, tax, or social benefits data) to patients’ healthcare records.

‘Socioeconomic information’ or ‘circumstances’ could include: markers of material living standards, such as housing, household income, or unemployment; indicators of ‘status’ like education level or occupation; or they could rely on broad categories or proxy measures such as ‘deprivation’, ‘socio-economic status’, or ‘class’. ‘Reasons’ could include any rationale, expressed in any terms, including feasibility, acceptability, importance, value, or ethics.

The following were not exclusion criteria and publications remained eligible for inclusion irrespective of: when and where they were published and in what language and what healthcare system they referred to; whether they included only particular patient groups (e.g. based on medical condition, geography, or demography); type of publication (e.g. editorials, opinion pieces, qualitative or quantitative studies, policy recommendations). We adopted this relatively inclusive approach to maximise the breadth of reasons: drawing on a broad range of literature and a range of philosophies and concerns. We did not limit our review to peer-reviewed studies, but included all published articles of any sort because our initial scoping of the literature suggested that there would be few if any intervention studies of socioeconomic enquiries in healthcare studies: thereafter we would be looking at hypotheses, theories, or opinions which might be published in letters, editorials, or opinion pieces as well as in a scientific studies.

Publications were *ineligible* and would *not* be included if they only discussed asking patients about socially-influenced health behaviours, such as alcohol consumption or smoking. And we would *not* include publications in which the necessity of collecting socioeconomic data or screening patients for adverse socioeconomic circumstances was *implied,* but never made explicit. (For example, a study concluding that ‘doctors should prescribe vitamins to children from low-income families’ implies the necessity of identifying low-income families. But the study would only be included if this was specifically acknowledged, for instance, if it stated ‘doctors should identify children from low-income families and prescribe them vitamins’.)

### Search strategy

The research question (‘What reasons have been given for asking, or not asking, patients about their socioeconomic circumstances in healthcare settings?’) was defined by authors AM and SZ. The search strategy was refined with the assistance of a medical librarian (NR). Six search databases were selected to access literature from disciplines of medicine and nursing (pubmed, embase, global health), ethics (Ethicsweb), social sciences (Web of Science), and psychology (PsychINFO). Our initial search strategy was relatively inclusive (high sensitivity), with greater emphasis on exclusion at the stage of screening. Database index terms proved unhelpful, so we constructed our search strategy using Boolean operations of non-controlled vocabulary for ‘Action’, ‘Object’, and ‘Setting/Staff’ (see Table 1).

**Table 1:** Terms used in literature search strategy

Action Terms	Include* OR record* OR document* OR using OR enquir* OR ask* OR collect* OR gather* OR monitor* OR screen*
Object Terms	Social OR socioeconomic OR demographic OR sociodemographic OR income OR wealth OR earning OR poverty OR poor OR depriv* OR “level of Education” OR “education level” OR “Educational attainment” OR “educational achievement” OR “academic achievement” OR numeracy OR “academic Attainment” OR literacy OR qualification* OR employment OR unemployment OR housing OR homeless OR homelessness OR accommodation
Additional Object Terms	Data OR details OR information OR determinants
Setting/Staff Terms	Healthcare OR “primary care” OR “general practice” OR “family practice” OR doctor OR doctors OR GP OR GPs OR nurse OR nurses OR clinician OR clinicians OR ‘clinical settings’ OR physician OR physicians OR “general practitioner” OR “general practitioner”

Trial searches were used to test search strings; ensuring searches captured relevant known references, but did not produce overwhelming numbers of results. The search strategy was repeated in the six selected databases during June 2016.

### Screening

Screening of search results was conducted by two authors (AM and AP) in two stages. First, a screening of title/abstracts; second, an in-depth screening of full-texts. At each stage, one author (AM) screened all references, while AP screened a 20% sample, with inter-rater reliability statistically confirmed using Cohen’s kappa coefficient.

### Snowballing

To identify further potentially relevant references, eligible publications were used in a four-stage snowballing process: checking each eligible publication’s own list of references; checking the first author’s other references on PubMed; checking the first 20 ‘related articles’ to the publication on google scholar; checking the publication’s citations on google scholar. Snowballing was done in September 2016. Snowballed titles were subject to full-text review.

### Data extraction and analysis

Data extraction was conducted by two authors (AM and AP). In each publication we identified reasons for collecting, or not collecting, patients’ socioeconomic information in healthcare settings, along with relevant challenges where authors identified these.

Details of each publication (full citation, year of publication, language, principle location of authors, the field and type of publication) were inserted into an excel table, along with the reasons contained in each text. Sometimes the reasons were clearly stated, at other times they required interpretation, as is consistent with the review of reasons methodology. To minimise bias, the two authors worked independently to identify and extract reasons, then group them according to those that were the same or very similar. Discrepancies were discussed and collaboratively resolved before reasons were categorised into themes.

## Results

The results of our systematic review are presented in the following order: publications included and their characteristics; published reasons FOR asking patients about socioeconomic circumstances; published reasons AGAINST; underlying principles referred to in the literature; challenges acknowledged to socioeconomic enquiries and data collection.

### Publications included

**Fig. 1:**
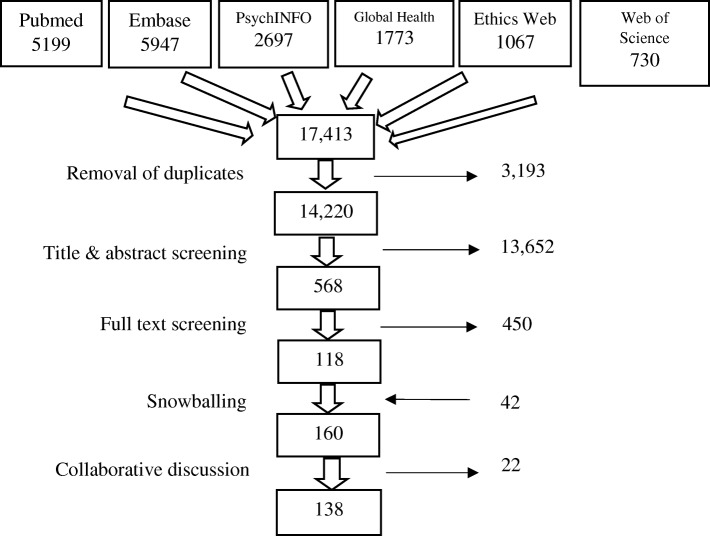
Outcomes of search and screening processes showing number of publications

The search strategy identified 17,413 results. Removal of duplications left 14,220 references. Title/Abstract screening resulted in 568 potentially eligible references. After full-text screening 118 references were considered to match the study criteria. These 118 were used in a snowball search that identified a further 42 references. The two screeners then reviewed and discussed all 160 references, agreeing to discard 22 as ineligible (see Fig. 1).

Ultimately, 138 publications met the inclusion criteria. Sixty-four of these publications were specifically concerned with our study question. In the remainder, the subject of collecting socioeconomic data was mentioned only peripherally. The earliest publication came from 1974, the latest from 2016. Though the number of publications addressing the subject increased almost year-on-year, this increase appeared consistent with the increase in publication numbers over time. Most publications were in English language, three were in French, one in Spanish. 72 publications were authored by individuals or teams in America, 20 in UK, 16 in Canada, 7 in France, 5 in Switzerland, 18 others were based in Australia, Belgium, Netherlands, New Zealand, Nicaragua, and Spain. Further details of publications are contained in Table 2.

**Table 2:** Characteristics of publications included in this systematic review

Publication Type	
Original research	67 (58 quantitative, 7 qualitative, 2 mixed)
Opinions, analyses, editorials, or letters	57
Practice or policy reports	10
Books or book chapters	4
Publication date	
1970–1979	4
1980–1989	1
1990–1999	12
2000–2009	34
2010–2016	87
Publication origin	
America	72
UK	20
Canada	16
France	7
Switzerland	5
Elsewhere	18
Publication field	
Clinical Medicine	
General medicine	34
Primary Care	28
Paediatrics	18
Other specialities	9
Public health /health policy	27
Nursing	4
Healthcareinformatics	7
Psychology	4
Ethics	3
Social work	2
Patient education	2

### Reasons FOR asking patients about socioeconomic circumstances in healthcare settings

Of the 138 publications meeting our inclusion criteria, 130 contained reasons for why patients’ socioeconomic circumstances *should* be enquired about in healthcare settings. From these 130 publications we extracted 275 separately stated reasons and grouped these into 15 broad reasons. We organised these reasons into three groups relating to: individual healthcare encounters; provision and organisation of health services; and population-level research and policy. The reasons and reason groups are shown in Table 3 and described in more detail below. Following our protocol, Table 3 includes all the relevant citations, we have not tried to identify the ‘main’ citation for each because this would be a matter of interpretation in such a disparate literature.

**Table 3:** Reasons for asking patients about their socioeconomic circumstances

Reasons	Citations
Reasons relating to individual healthcare encounters	
Clinicians can refer patients to social resources	[10, 16–38]
Clinicians can engage directly with patients’ social needs	[18, 34, 39, 40]
Clinicians can acknowledge patients’ socially-determined risk of disease (specifically cardiovascular disease risk)	[41–49] ( [16, 23, 35, 50–52])
More clinical resources can be allocated to patients facing adverse social conditions	[26, 42, 53–57]
Clinical management plans can be adapted to patients’ socioeconomic context	[11, 16, 32, 38, 58–70]
Clinicians can better understand non-adherence to management plans	[26, 58, 66, 71, 72]
Communication and relationships can be improved between patients and clinicians	[54, 73–76]
Patient preferences	[27, 77, 78]
Reasons relating to health service provision and organisation	
Healthcare use by different socioeconomic groups can be better monitored	[26, 31, 43, 79–89]
More healthcare resources can be allocated to populations with greater need	[35, 90–93]
Healthcare services can be better adapted to population needs	[10, 16, 23, 26, 32, 42, 43, 60, 68, 87, 92, 94]
Deprivation payments can be more accurately allocated	[55, 82, 90, 95–97]
Reasons relating to population-level research and policies	
Health research can be improved	[13, 16, 35, 45, 46, 60, 73, 82, 89, 98–104]
Public health policies can be better-informed	[10, 23, 32, 42, 44, 57, 66, 81, 105–107]
Health and social care can be better integrated	[29, 31, 101]

### Reasons relating to individual healthcare encounters

The most common reason for asking patients about their social circumstances was to enable social difficulties to be identified and addressed. Mostly, this would be by referral from the healthcare setting to an outside resource. [10, 16–37] Physicians, it was said, would welcome the ‘unburdening’ of having to respond to patients’ social needs. [38]

But some authors suggested healthcare providers might themselves play a more active role in responding to patients’ social difficulties. For example, directing patients to food banks, [34] writing advocacy letters, [18] supporting their employment, [40], or ensuring the housing tenancies of vulnerable patients. [39]

Several authors observed the relevance of socioeconomic enquiries to the clinical assessment of disease risk. [41, 44–46, 48, 49] New Zealander Peter Crampton in 2000 wrote:

‘It is remiss that low socioeconomic status – a strong risk factor for poor health – is not usually included in our explicit and implicit checklists when reviewing patients. Rather, we tend to focus on behaviours (e.g. smoking, diet, exercise), demographics (age, sex) and genes (family history).’ [42]

Specific associations have been noted between unemployment and mental health, [43] and low levels of maternal education and newborn hearing impairment. [47] Screening for these conditions could be better targeted if socioeconomic information appeared in clinical records.

Six papers (from different authors) argued that a reliable socio-economic marker should be incorporated into cardiovascular risk assessment and management. [16, 23, 35, 50–52] If a person’s socioeconomic circumstances increased their risk of cardiovascular disease, it was suggested, more effort might be made to control their blood pressure or cholesterol, or to influence their smoking or exercise habits. Notably, none of these papers remarked on the ethics of endeavouring to ameliorate the adverse effects of unhealthy social conditions through greater medical management or individual lifestyle modification.

More generally, ‘targeting resources’,such as time, to individuals whose socioeconomic circumstances put them at ‘high risk’ was advocated. [42, 55–57] More clinical time could be allocated to deprived patients to deliver preventative care, [26] provide more detailed explanations and advice, [53] and improve physician-patient relationships. [54] The justice-based reasoning underlying these proposals was sometimes up-turned. For example, clinical ethicists Payot and colleagues have asked whether infants from families of low socioeconomic status should be identified because it made little sense to invest very scarce clinical resources when low socio-economic status is a risk factor for high mortality. [108]

In 1976 Barclay et al. in Scotland observed that social knowledge is necessary for doctors to manage their patients’ problems. [109] The relevance of socioeconomic context was later highlighted in the management of specific health problems including diabetes, [32, 69, 110] rheumatoid arthritis, [61, 63] and psychosis. [62] The specific socioeconomic context of vulnerable housing is a particular challenge: ‘Lack of stable housing can affect a patient’s ability to store and take medications, to care for wounds properly, and to abstain from or limit physical activity during recuperation.’ [68] It was said that acknowledging patients’ socioeconomic contexts could enable improved treatment planning [11, 58, 59, 64, 66] and shared decision-making. [16, 58, 60] It could also improve the effectiveness [65] and outcomes of healthcare, [67] while reducing costs [38] and improving patient satisfaction. [58]

‘Non-judgemental care’ could be facilitated by better understanding of patients’ social contexts. [111] Australian primary care physicians Furler and Young specifically cautioned against ‘blaming patients for apparent “non-compliance”’; acknowledging that socioeconomic circumstances might explain patterns of healthcare engagement. [26] In America, Behforouz and colleagues also recognised that ‘nonadherence to treatment plans, missing of appointments, or failure to fill prescriptions’ might reflect a patient’s socioeconomic context. [58] Healthcare costs and expenses incurred indirectly in following treatment plans obviously vary between different healthcare systems and represent an obvious barrier to compliance. [66] Less obvious socioeconomic barriers to compliance include lower levels of education [71] and inflexible work schedules. [72]

Writing in 1974, Murray and colleagues in England had suggested that discussions about social problems could ‘establish the basis of a good doctor-patient relationship’. [76] Later authors agreed that knowledge of patients’ social difficulties would enhance relationships [74] and improve communication. [73, 75] Since patients who experience poor socioeconomic circumstances have poorer experiences of healthcare encounters it has been suggested that greater effort should be made to communicate with these patients in particular. [54]

Three studies that investigated patient opinions about socioeconomic enquiries in healthcare settings found generally positive attitudes. The participants of focus groups conducted among low-income primary care patients in America said they appreciated healthcare professionals ‘being sensitive to their financial concerns’. [78] A questionnaire study undertaken at an American children’s hospital found ‘parents have favourable attitudes toward asking for and receiving assistance for these needs from their child’s provider.’ [27] Another questionnaire study conducted among a primary care population in Canada reported that ‘respondents felt that asking about poverty-related issues in primary care is important’. [77]

### Reasons relating to health service provision and organisation

In the 1990s it was proposed that the routine collection of patients’ socioeconomic information would enable UK general practices to better define the needs of their population and evaluate service delivery. [43, 82] More recently, gathering patients’ socioeconomic data has been said to constitute ‘part of good quality primary care data’, [80] to be vital for audit, [81, 85] especially audit of service uptake [26, 89] and impact, [31] as well as for ‘monitoring’ inequalities in healthcare delivery or access. [79, 83, 84, 86–88] For example, Moser and colleagues have suggested that ‘the routine collection within general practice of additional sociodemographic information would aid monitoring of inequalities in screening coverage and inform policies to correct them.’ [84] Similarly, preventative strategies, including childhood immunisations, might be better resourced and targeted because these activities ‘are known to reach those in poor socioeconomic circumstances less well’. [35, 92] Socioeconomic data could then be used to direct service provision toward ‘areas of highest need’. [90, 91, 93] The data could also enable healthcare providers to better understand the particular needs of populations and adapt services to meet those needs. [16, 42, 43, 60, 68, 87, 92, 111] Examples of adaptations included: initiating new services or support groups, [16, 26] adjusting clinic hours to improve access, [94] and developing decision-support systems for chronic disease. [32] Other suggestions have been the co-location or improved coordination of health and social services. [10, 23]

Another reason for recording the socioeconomic demographics of the general practice population, is to provide a more valid basis for the calculation of deprivation payments. In Britain and New Zealand these payments increase resources for practices working with deprived populations. [82, 90, 97, 112] In America too, patients’ socioeconomic data could be used to adjust physician performance or outcome measures; providing recompense to doctors whose patients are poorer and less healthy. [95, 96] Thus, ‘Failure to account for patient SES [socioeconomic status] may penalize physicians caring for poorer patients’, [55] and ‘may encourage selective enrolment of more affluent patients in an effort to improve performance ratings’. [95]

### Reasons relating to population-level research and policies

Several authors suggest that recording socioeconomic data in healthcare records would provide new opportunities for health research. [13, 16, 73, 99, 102, 104] The linked health and socioeconomic data would evidence the nature and extent of health inequities [35] and also help researchers to study the aetiological mechanisms of social health determinants, [45, 46, 60, 82, 89, 98, 103] and identify the population subgroups in which they are most relevant. [101] The data might improve understanding of how social factors affect disease incidence, [103] individual health outcomes, [100] and outcomes of clinical care. [60] Even for research not specifically concerned with social factors, understanding their role as potential confounders could be illuminating since they may act as important confounders.

Public health policies could be better designed and their effects better assessed using linked health and socioeconomic data. [42, 57, 60, 66, 105–107, 113] The data could be utilised at the local and national level, [32, 44] in the planning and evaluation of wider policies, [10, 81] including transport and food policies. [60]

Identifying social health determinants in healthcare settings could create a basis for ‘the integration of social services and medical care’, [31] representing a ‘high-value benefit to the health care system’, [29] enabling ‘better and more efficient health care’. [101] Ultimately, addressing social needs might reduce population healthcare costs. [114]

### Reasons for *NOT* asking patients about their socioeconomic circumstances in healthcare settings

Of the 138 publications meeting our inclusion criteria, none argued explicitly that socioeconomic data should NOT be collected in healthcare settings. Five papers expressed scepticism about the value, feasibility, or efficiency of collecting patient data. [115–119] Three other papers did not mention socioeconomic data collection specifically, but argued more broadly that social health determinants lay outside the remit of the medical profession. [100, 120, 121] Reasons contained in these eight papers were grouped as shown in Table 4.

**Table 4:** Reasons for NOT asking patients about their socioeconomic circumstances

Reasons	Citations
Reasons relating to individual healthcare encounters	
Socioeconomic enquiries will conflict with clinical tasks	[121]
Socioeconomic enquiries will overburden clinicians	[117, 121]
Socioeconomic enquiries might foster patient distrust	[118]
Reasons relating to data	
Data collection would be of poor quality, especially among deprived groups	[116–118]
Existing sources of socioeconomic information are adequate	[115, 117–119]
Limits to medicine	
Social health determinants lay outside the remit of the medical profession	[100, 120, 121]

Clinical staff asking patients about their socioeconomic circumstances could be a source of potential harm, it was suggested. These enquiries would either occur at the expense of clinical tasks, to the potential detriment of patients, or in addition to clinical tasks, to the detriment of ‘overworked’ staff. [117, 121] Socioeconomic enquiries might also foster patient distrust, particularly around potential uses of the information. [118]

The value of the data collected might be limited, especially as a tool for monitoring inequalities, because the most deprived patients would be least likely to provide the socioeconomic information requested. [117] This would be due to higher levels of distrust, [118] or because of less frequent encounters with healthcare facilities. [116]

It was said that informal appraisal of patients’ socioeconomic factors already occurred in clinical settings and this was adequate for clinical purposes. [117] Meanwhile, for the purposes of monitoring and public health, existing area-based deprivation data was said to be sufficient. [115, 118, 119]

Socioeconomic influences upon health were said to lie outside the core of individual patient care and hence beyond the ‘domains of professional obligation’. These domains could be empirically defined according to ‘the feasibility and efficacy of physician involvement’; [100] clinicians having neither ‘expertise or resources for this work’. [121] Alternatively, professional domains could be normatively construed, with it being suggested that ‘doctors act as the guardians of an enclosed space where socioeconomic status ought to play no role.’ [120]

### Principles cited in relation to socioeconomic enquiries

The claims about limits to the professional domains of doctors seen in the preceeding section suggest the contingent nature of discussion around socioeconomic data collection. Many authors cited underlying beliefs or principles that could be contested, attributed variable importance, or variably interpreted – see Table 5.

**Table 5:** Underlying principles cited in relation to socioeconomic enquiries

Principles cited	
Reducing health & healthcare inequalities	[26, 29, 42, 57, 61, 63, 72, 73, 83, 84, 86, 93, 94, 122–127]
Duties and potential roles of doctors	[32, 53, 73, 100, 128, 129]
Patient-centeredness	[13, 20, 26, 27, 72, 127, 130–137]
Evidence-based medicine	[67, 138]
Relevance of measurement and data to healthcare performance	[14, 43, 86]

Despite assertions above about the limited role of doctors in relation to inequalities, reducing socioeconomic inequalities in health is frequently cited as an important objective. [29, 57, 61, 63, 72, 83, 93, 94, 123, 125, 127] So too is reducing socio-economic inequalities in healthcare experience, [73] access and delivery, [42, 57, 86] screening programs [84] and prevention strategies [26]. Monitoring healthcare inequalities and implementing steps to reduce them has been described as a marker of healthcare ‘quality’ [83, 122, 124, 126] as well as an ‘ethical obligation’. [86]

Other ethical duties raised in the literature included the doctor’s duty to acknowledge patients’ socioeconomic circumstances during clinical decision-making [129] and ‘to search actively for socioeconomic barriers to effective treatment.’ [53] Some authors thought doctors had public duties too. As a ‘key witness of social inequalities in health’, [73] the primary care doctor could bring ‘information and professional authority’ to public debate. [100] The President of the College of Family Physicians of Canada, suggested in 2012 that, individually and collectively, physicians had a role and responsibility to raise awareness and advocate for action on social health determinants. [128] This public role did not necessarily require doctors to become more outspoken: discussing socioeconomic circumstances with patients and documenting these could be sufficient, for by doing so, doctors would ‘publicly recognize the role that income instability, low educational attainment, inadequate housing, and food insecurity currently play in disease development’. [32]

Patient-centredness was another principle that was often cited in relation to socioeconomic enquiries. In 1977, Zander, a London GP, wrote that seeing the patient and their problem in the context of their social situation was an intrinsic part of general practice. [136] In 1989, Henk proposed that socioeconomic difficulties should be included in Problem-Orientated Medical Records. [137] Latterly, a social perspective and acknowledgement of socioeconomic realities has been part of approaches variously described as ‘biopsychosocial’, [72, 133, 139] ‘client-centred’, [127] ‘patient-centred’, [13, 20, 134] ‘patient-tailored’, [131] ‘whole-person’, [132, 135] or sensitive to ‘context’ [26] or ‘social milieu’. [27] However, physician Susan Bernheim and colleagues have expressed concern that while tailoring healthcare to patients’ financial constraints may be appropriate, it is not the same as adapting care to patients values or beliefs (‘as is central to most definitions of patient-centred care’). They suggest that there may be a conflict between financially-constrained care and patient-centred care. [130]

Evidence-Based Medicine (EBM) was also raised in the literature as a potential deterrent to considering a patient’s socioeconomic circumstances, one author suggested that ‘taking into account socioeconomic circumstances in treatment options … may create conflict with EBM-directed solutions based on biomedical efficacy’. [129] But others disagreed. They suggested that social factors routinely influence clinical decision-making, and ‘true EBM’ required the role of these non-clinical factors to be recognised, understood, and acknowledged. [138] [67].

Ultimately, some authors acknowledged that decisions about socioeconomic data collection would reflect priorities in healthcare. Those priorities would also determine how healthcare performance was measured, and whether this included measurement of socioeconomic equity. More than two decades ago, Fleming and colleagues suggested that socioeconomic data collected from patients ‘may in future contribute to the design of performance indicators in general practice.’ [43] Rosen quoted the management psychologist Mason Haire: ‘Those parts of the work that are not considered important enough for regular measuring are easily disregarded’. [86] Or, as Wray et al. suggested, ‘as the old saying goes, “you cannot manage what you don’t measure”.’ [14]

### Surmountable challenges to asking patients about their socioeconomic circumstances in healthcare settings

Many authors who proposed that patients should be asked about their socioeconomic circumstances also acknowledged the challenges involved. Some of these challenges overlapped with the reasons put forward for *not* asking patients; including potential conflicts with clinical tasks, questions about the acceptability of socioeconomic enquiries and the accuracy of the data that would be obtained. However, these were not seen as reasons for not asking patients about their social circumstances, but as challenges to be overcome.

Lack of time was frequently cited as a barrier; [16, 19, 21, 28, 34, 41, 98, 123, 131, 140–142] presumably reflecting a perception that other tasks have higher priority. Also, staff might believe patients’ socioeconomic circumstances lie beyond their remit [17, 26, 34, 60, 66, 143] or influence. [19, 20, 41, 58, 131, 144] They might not feel comfortable talking to patients about these subjects, [41, 58, 79, 131] or they might be concerned about how patients would respond. [14, 92] Staff may lack knowledge about how to broach social issues with patients, [142] about relevant assessment tools, [21] about how to respond when social problems were identified, [36] and about resources to which patients might be referred. [19, 21, 24, 28, 141] Some of these issues might be attributable to the lack of training of health professionals on social health determinants [17, 24, 53, 58, 62, 81] and how these can be incorporated into clinical care. [19, 21, 41, 123, 144]. Training on social health determinants, on the rationale and procedure of socioeconomic data collection, on the identification and management of social problems, [36] and local referral resources were all advocated, [18, 27] as well as training on how to discuss socioeconomic issues sensitively with patients [24] and how to communicate the rationale for the discussion. [14, 101]

The accuracy of information that patients might provide and its adequacy for decision-making were questioned. [66, 68, 86, 92, 98, 145] Self-reported socioeconomic information may be influenced by social desirability bias, stigma, or self-interest. [32, 56, 131, 140] Current classification systems used in healthcare settings are poorly suited to socioeconomic details. [67, 92] The difficulty of entering, accessing, and sharing socioeconomic information using current electronic healthcare records was raised . [16, 19, 48, 60, 70, 98, 103, 115, 146] Some of these technical issues could be addressed by improvements in health information technology and Electronic health record design. [23, 31, 35, 36, 41, 80, 126, 145, 146] Authors also recommend that tools for socioeconomic data collection should be standardised, practical, validated, and evidence-based with no intellectual property restrictions. [18, 22, 92, 94, 99, 118] New guidelines and policies would help. [29, 80, 124, 146] So too would new data collection processes that are sensitive and culturally acceptable, [18, 26] time-efficient, [27] and integrated into clinical workflows, [36] while imposing minimal burden on clinicians, practices, and patients. [11] There are concerns about who should ask the questions and how often, [16] with suggestions that non-clinical members of the healthcare team might collect the data [21, 123] or patients might self-complete forms, which could be on-line. [11, 13, 16, 19, 56, 132, 141, 145, 147]

Although referral to social resources was the most often-cited reason for asking patients about their socioeconomic circumstances, it was acknowledged that such resources were limited [22] and too often unreliably funded. [114] Healthcare staff would need to be aware of local social resources [24, 27, 73] and feel able to make referrals. [41] Compiling a list of community resources could be helpful, [25] or co-locating healthcare and community resources in the same physical space. [29] Some authors contended that asking patients about socioeconomic problems might be inappropriate if there were no relevant resources available, [22, 28] while acknowledging the danger of a cycle of inaction, for if enquiries are not made and needs are not identified, resources are unlikely to appear.

‘Privacy’ concerns were commonly expressed. [10, 13, 39, 60, 66, 83, 86, 96, 113, 122, 126] But ‘privacy’ was variously construed. It was seen by some authors as a concern of patients being asked questions about private matters, [66, 96, 122] by others as an issue relating to data storage, with ‘privacy and security’ sometimes cited as parallel concerns. [39, 83, 113] Privacy was also at stake in the use of health records for purposes beyond individual patient care, [60] for example, ‘use of the information by insurers to increase a patient’s premium or by health systems to avoid high-risk patients’. [16]

Some authors thought that patients might not feel comfortable discussing their socioeconomic circumstances in healthcare settings, [14, 26, 56, 79, 91, 94] possibly due to concerns about confidentiality, stigma, or data use, [66, 83] or even a negative impact on their care. [83, 126] Low public awareness about health inequalities was said to be relevant [57, 83] suggesting that patients might need to be informed about why they were being asked about their socioeconomic circumstances. [14, 124] This message was said to be especially relevant to vulnerable groups. [118] However, it was emphasised that, to reduce concerns about stigmatization, *all* patients should be asked about their socioeconomic circumstances. [21] Engagement of community representatives or advisory groups was also proposed. [25]

Although there were concerns about the impact of socioeconomic enquiries on relationships between patients and providers, [57, 66] others pointed out that these relationships may be less important than potential interventions and patient health benefits. [111]

Finally, institutional lack of commitment, [146] organizational inertia and lack of leadership, [122] as well as the lack of an ‘operational approach to get from theory to practice’ [36] were said to hinder uptake of socioeconomic enquiries in healthcare settings. The lack of financial incentive was also suggested as a barrier. [31, 92, 114] Accordingly, socioeconomic data collection might be motivated by stronger financial reimbursement strategies, [10, 11, 16, 58, 60, 80, 101, 122] as well as improved leadership, [145] greater political will, [83] and more effort to shift medical culture. [11] Several authors also noted the importance and impact of changes in American legislation and the resulting recommendations of the Institute of Medicine. [23, 101, 144, 147]

## Discussion

This is the first published summary of literature addressing the subject of asking patients about their socioeconomic circumstances. Our focus on the reasons for and against socioeconomic enquiries in healthcare settings is timely at a moment when efforts are being made to implement the practice in some contexts. Our broad and inclusive systematic search strategy has identified a breadth of reasons in a diversity of publications from a range of places and times. We have presented our findings in a way that will enable those concerned with healthcare policy and practice to grasp the relevant issues and the arguments that have been put forward. It should be noted that in many instances the stated reasons for or against socioeconomic enquiries were based on hypothesised outcomes, reflecting the lack of published intervention studies. Our review of reasons provides future researchers with ideas for testable hypotheses when routine socioeconomic enquiries are adopted in different settings.

Limitations of our work include the possible bias of search databases toward English language results (though we did not restrict our searches by language) and the possibility that our search terms may not have captured all eligible publications (particularly since there are no relevant specific key words and the subject is variously and inconsistently phrased using descriptive terminology). Also, this is a summary of published reasons, and it is important to acknowledge that not all reasons may appear in print, whether on account of their sensitivity, or because explicit statements of reasons are not usually required to uphold existing practices. The fact that we found more reasons and publications suggesting why socioeconomic enquiries *should* occur may reflect a situation in which they mostly *do not* occur: so far most writing on the subject has been by advocates of change; if changes to practice occur, or are increasingly considered and discussed, then more opposition may appear in the literature. At this stage however, it may be supposed that some degree of research or publication bias exists. A further limitation of our work might appear to be the absence of a ‘quality assessment’ of the reasons identified or the sources in which they appear. This is a criticism of the review of reasons methodology to which we would respond by pointing out that a reason which is highly relevant and valid to one context, or indeed to one reader, may be irrelevant or invalid to another: the ‘quality’ of any reason will be contextual and the judgement will be inherently subjective. Instead, we have sought to describe and where necessary explain reasons so that their relevance and persuasiveness can be evaluated according to the setting in which they may be applied and the values of those who would apply them.

Illustrating the above point, different reasons, with different mechanisms of change, appear to have motivated socioeconomic data collection in the very different healthcare systems of America and Canada. Broadly speaking, in America it seems that the principle reason for promoting socioeconomic enquiries has been to control healthcare costs while improving health outcomes, and the mechanism has been top-down legislation and financial incentivisation. By contrast, in Toronto, Canada, the main reason has been concern for healthcare equity and the change has been driven by determined bottom-up efforts. In the UK, different reasons again have been put forward in favour of socioeconomic enquiries in healthcare settings. The earliest papers included in our review date from 1970s Britain (a time when UK general practice was expanding and professionalising) and their authors expressed concern for comprehensive record keeping. [76, 109, 136] During the 1990s in Britain, the issue of asking patients about their socioeconomic circumstances was again discussed, this time in the context of debate about ‘deprivation allowances’ that were intended to reimburse UK practices that worked with deprived patient populations. Central to this issue was the question of whether the relative deprivation of practice populations should be gauged using area data (based on averages of census-derived data applied to populations within geographical areas) or, as was advocated (unsuccessfully, at the time), using data based on the collection of information from individual patients within each practice population. [43, 82, 87, 97, 112] Ultimately, area-based, census-derived, composite indices of ‘deprivation’ continue to be used to determine a component of UK general practice funding.

The relative merits of area-level and individual-level socioeconomic data has been a recurring debate in some countries. While the suitability of each data set will depend on the purpose to which it is to be put, several papers in our review noted the relative weaknesses of geographic area data. [73, 77, 104] Area-level data may adequately describe a practice population overall, but it cannot reliably be used to infer characteristics of individuals within that population. Specifically, it cannot be used to identify individuals who experience deprivation, whether for purposes of clinical care, research, or monitoring and service evaluation. [148–151] Moreover, the composite outcome scores of area-based deprivation indices tend to obscure the lived experiences of deprivation, and do not identify where or how to intervene, nor on which components of deprivation.

Despite recent developments in North America, we did not find increasing consideration being given to the subject of socioeconomic enquiries in UK publications, either to uphold or challenge current practices. Quite the opposite: we found that while the global trend over time was for increasing numbers of publications to address the subject, this trend was absent in UK publications. Globally, in the period up to and including 1999, 17 publications addressed the subject, and of these, 11 (roughly two thirds) were from the UK. Yet of the 121 articles published since 2000, most [69] were from America, a smaller number [15] from Canada, and only 9 (less than 10%) were from the UK. The apparently diminishing UK interest in the issue and the absence of changes to UK practice in this area may again suggest the contextual contingency of the reasons included in this review.

While the question of whether to ask patients about their socioeconomic circumstances has been largely ignored in the UK lately, in America and parts of Canada, the question has moved on to how, practically, these enquiries can best be made. It has been implied or explicitly stated that primary care may be the best context for socioeconomic enquiries. [58, 128] Yet there is no consensus on who should ask the questions. [25, 70] The American Institute of Medicine has emphasised that a member of the clinical team should ask the questions, though it has been pointed out that it did not need to be a physician. [21] In Canada, a survey of 1,306 people showed that 29% of participants would prefer to disclose socioeconomic information to a doctor, 22% to a clerk, and 20% on a form in a clinical setting. [83] The feasibility of using an electronic tablet to gather socioeconomic information in a clinical setting has also been demonstrated. [57] Socioeconomic enquiries via the internet or mobile technology have also been proposed, [11, 19, 79, 132] though in the Canadian study this method had the lowest approval.

Various suggestions have been made for exactly which questions should be asked, with a variety of multiple-question schedules described. [14, 16, 28, 45, 106, 144] Single-question screeners have also been advocated. In Switzerland, Bodenmann and colleagues found that the single question ‘Did you have difficulties paying your household bills during the last 12 months?’ identified patients at risk of forgoing healthcare for economic reasons (sensitivity 74.1%, specificity 79.9%). [131] In Canada, Brcic and colleagues found that the question ‘Do you (ever) have difficulty making ends meet at the end of the month?’ was a good predictor of poverty (sensitivity 98%, specificity 60%). [77] The issue of how frequently the enquiries should be repeated remains unresolved. [25, 43, 57, 86] Further research was said to be required on data collection tools, [132] clinician attitudes, [141] and the impact of enquiries and ensuing interventions. [19, 21, 56, 146, 147] Future research might also further explore the attitudes of patients toward socioeconomic enquiries and their understanding of its purpose and value.

## Conclusion

The published literature contains many varied reasons for asking patients about their socioeconomic circumstances. These reasons have appeared with increasing frequency, in a range of literature, and have been applied to a range of settings and healthcare systems. It has been said that recognising and responding to social health determinants may improve health outcomes for patients and populations, while reducing demands upon healthcare and enabling more cost-effective health services. It has also been suggested that incorporating patient socioeconomic information into service evaluation and, for example, monitoring socioeconomic variation in incidences of late cancer diagnoses, hospital referral rates, or engagement in health protection strategies, could become a powerful means of promoting healthcare equity. Consistent with evidence-based principles, it has been claimed that replacing area-level proxy indicators of deprivation with individual-level socioeconomic information would support more accurate research, enabling improved healthcare, as well as improved understanding of the extent and aetiology of health inequalities and the impact of interventions and policies. However, concerns have been expressed around acceptability, data quality, and the limits to the role of clinical teams. Also, and importantly, it should be noted that the reasons stated for and against socioeconomic enquires are mostly based on hypothesised outcomes, rather than evidence. So while developments in America and Canada may set new precedents, and demonstrate the feasibility and acceptability of socioeconomic enquiries, further research is needed to show whether the hypothesised benefits are real. Our review has described the existing reasons that might influence potential changes to policy and practice in this area. It has also highlighted the conjectured outcomes that could be tested by future research to provide robust evidence for implementation.

## Data Availability

The protocol used in the literature search is included in a separate file. Any further information or material is available from the author on request.

## Abbreviations

GP: General Practitioner (primary care physician)
UK: United Kingdom of Great Britain and Northern Ireland
